# Megameatus intact prepuce: a systematic review of surgical techniques and long-term outcomes

**DOI:** 10.1007/s00383-024-05898-4

**Published:** 2024-12-03

**Authors:** Abubakr Elawad, Ahmed Haroon, Jamil Ahmad, Jude Alsbeti, Sami Cherigui, Seem Arar, V. V. S. Chandrasekharam, Tariq O. Abbas

**Affiliations:** 1 Pediatric Urology Division, Surgery Department, Sidra Medicine, Doha, Qatar; 2 Urology Department, Hamad Medical Corporation, Doha, Qatar; 3 Pediatric Surgery Division, Department of Surgery, Hamad Medical Corporation, Doha, Qatar; 4 Pediatric Urology, Pediatric Surgery & MIS, Ankura Hospitals for Women and Children, Hyderabad, India; 5 College of Medicine, Qatar University, Doha, Qatar; 6 Weill Cornell Medicine-Qatar, Doha, Qatar

**Keywords:** Megameatus intact prepuce, Hypospadias, Urethral meatus, Surgical management

## Abstract

Megameatus intact prepuce (MIP) presents with diverse phenotypes that complicate the management of this rare but complex hypospadias variant. Current data on optimal treatment methods and patient outcomes are sparse, unintegrated, and therefore challenging to implement clinically. A comprehensive systematic review of the existing literature on MIP was conducted according to PRISMA guidelines. Electronic databases including PubMed, Embase, and Scopus were searched for relevant articles published up to [2024]. Key aims were to assess the safety and efficacy of different surgical interventions, and synthesize corresponding outcomes reported in the literature. The search yielded 18 articles meeting the inclusion criteria, representing a total of 524 enrolled patients across multiple geographic regions. Diagnosis of MIP typically involves clinical examination, imaging studies, and urological evaluation. Surgical management options included preputial reconstruction, urethroplasty, and meatal advancement with glanuloplasty. Reported outcomes varied, with success rates ranging from [77.1–100%]. Long-term follow-up data on functional and cosmetic outcomes were limited. Megameatus intact prepuce presents diagnostic and management challenges due to low prevalence and variable presentation. This systematic review presents a current understanding of MIP diagnosis, surgical techniques, and patient outcomes. Future studies should assess the long-term functional outcomes of different surgical approaches, and investigate the underlying genetic and environmental factors contributing to the diverse clinical manifestations of MIP.

## Introduction

Megameatus intact prepuce (MIP) is a rare congenital anomaly characterized by key anatomical features that are distinct from typical presentations of hypospadias. These include an intact foreskin, a wide fish mouth-like urethral opening, a deep navicular fossa, a broad shovel-shaped glans, and an absence of ventral curvature [1]. Diagnosis and management MIP are extremely challenging, and post-operative outcomes remain poorly documented [2, 3]. Individual case reports and small series have contributed to the current understanding of MIP, but a comprehensive synthesis of the existing literature on the clinical management of this condition is lacking [4].

MIP accounts for approximately 3–6% of all hypospadias repairs [5], although the exact incidence of this condition in the wider population remains unknown. Some patients with MIP may not be identified or might not seek medical attention, particularly if the urethral anomaly is not perceived as clinically significant. However, with an increasing prevalence of routine physical examinations, rates of MIP detection are now rising.

MIP is theorized to result from incomplete fusion of the urethral folds, leading to an open distal urethra and normal prepuce formation. Over-canalization or persistent distal splitting of the ventral urethra may cause disunion of the glans, leading to the characteristic large meatus and deep glans cleft [6]. While other hypospadias variants have been linked to environmental factors such as in vitro fertilization (IVF) and estrogen exposure, no definitive associations have been identified for MIP. Additionally, the genetic etiology of MIP remains under-investigated compared to other hypospadias variants. Further research is therefore needed to explore potential causative factors and better understand the mechanisms underlying this condition.

In this manuscript, we present a systematic review of the current literature on MIP, aiming to provide a comprehensive overview of clinical presentation, associated anomalies, diagnostic modalities, management strategies, and long-term outcomes. Key objectives were to describe the clinical features and phenotypic variants of MIP, review contemporary approaches to case management, and discuss the long-term implications and quality of life outcomes for affected individuals (including potential complications and sequelae).

## Methodology

### Search strategy

A comprehensive search was conducted using electronic databases including PubMed/MEDLINE, Embase, Cochrane Library, and Web of Science. The search strategy utilized a combination of relevant keywords and medical subject headings (MeSH terms), such as “megameatus intact prepuce hypospadias”, “hypospadias with intact foreskin”, and related terms. The search was limited to articles published between June 1989 and Feb 2024, with cohorts including a minimum of 5 cases. Hand-searching of reference lists from included studies and relevant reviews was also performed to identify additional sources.

### Study selection criteria

**Fig. 1:**
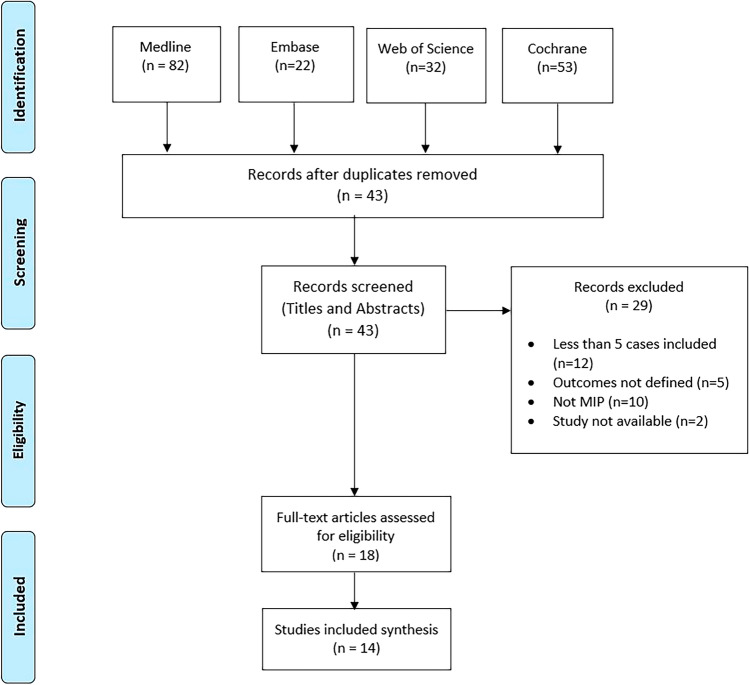
PRISMA flowchart

Studies were included in this systematic review if they met the following criteria: (1) focused on megameatus intact prepuce hypospadias, (2) included human participants of any age, (3) provided clear descriptions of the study population, interventions, and outcomes, and (4) were available in English or translations were available. Two independent reviewers screened titles and abstracts of identified records for relevance, followed by a full-text screening of potentially eligible studies. Discrepancies between reviewers were resolved through discussion to reach consensus (Fig. 1). Any studies not meeting the inclusion criteria were excluded.

### Data extraction, synthesis and analysis

Data were extracted from included studies using a standardized form, including key characteristics (e.g., author, publication year, study design), participant demographics, interventions/exposures, comparators, outcomes, and key findings. Quality assessment of included studies was conducted using The Newcastle–Ottawa Scale (NOS), with any disagreements resolved through discussion. Narrative synthesis was performed to summarize findings across included studies. Due to the significant heterogeneity of studies, a meta-analysis to estimate pooled effect sizes was not conducted.

### Quality assessment

Following the identification of eligible studies, two reviewers independently assessed the methodological quality of each report using NOS. Disagreements between reviewers were resolved through discussion or consultation with a third reviewer to reach consensus. The NOS tool was customized to suit the specific study design and objectives of this systematic review. NOS assigns stars to individual study components based on predetermined criteria, then tallies the stars to calculate an overall quality score for each report. Studies were categorized as poor (0–3 stars), fair (4–6 stars), or good quality (7–9 stars) based on predefined criteria assessing the selection of study groups, comparability of groups, and determination of outcomes. Studies rated as “poor” were considered for inclusion after careful consideration of their limitations, while studies rated as “fair” or “good” were accorded higher weight during data synthesis and interpretation. Sensitivity analyses were conducted to explore the impact of study quality on the overall findings of this review. The results of the quality assessment were also tabulated. Descriptive statistics, including mean scores and ranges, were used to summarize the methodological quality of included studies. Subgroup analyses were performed to examine whether study quality influenced the magnitude or direction of effect estimates.

### Reporting guidelines

This systematic review was conducted in accordance with the Preferred Reporting Items for Systematic Reviews and Meta-Analyses (PRISMA) guidelines.

### Statistics

Methods used in this study were appropriate for the data format and research questions being addressed. Frequencies and percentages were used to describe the categorical variables, while means and standard deviations were used to describe numerical variables. A Sankey diagram was used to visualize associations between hypospadias type, surgical technique, and complications status. Since none of the studies included essential statistical measures like odds ratio (OR), relative risk (RR), or Chi-squared (χ2) tests for comparison, it was not possible to conduct a heterogeneity test or perform a meta-analysis.

## Results

### Study characteristics

Included studies comprised a variety of designs, including case reports, case series, retrospective cohort studies, and prospective analyses (publication dates ranged from 1989 to 2024). In total, this amounted to 524 patients enrolled across multiple geographic regions (5 studies originated in North America, 1 in Europe, 10 in Asia, and 2 in Africa; Table 1).

**Table 1:** List of included MIP studies

	Author	Year	Number of cases	Age of patients (months)	Procedure used	Complication rate (%)	Follow up duration (months)
1.	Duckett JW et al*.* [5]	1989	14	6–39	Pyramid procedure	0	2–36
2.	Belloli GP [7]	1993	34	24–168	Cutaneous advancement procedure (CAP)	0%	5–6
3.	Hill GA [8]	1993	37	7–96 (12)	Pyramid repair (*n* = 35) Thiersch Duplay repair (*n *= 2)	5.4% Pyramid: Fistula (*n* = 2)	3–36 (16.5)
4.	Nonomura K [9]	1998	9	24–60 (42)	Mathieu (*n* = 5) Onlay urethroplasty (OUPF II) (*n* = 4)	0%	7–47
5.	Peretz D [10]	2003	21	0–252 (26.4)	Mathieu (*n* = 12) No intervention (*n* = 9)	Not reported Only discussed pseudo-iatrogenic hypospadias	
6.	Bar-Yosef [11]	2005	24	6–60 (18.5)	MAGPI (*n* = 2) GAP (*n *= 11) TIP (*n* = 11)	16.7 GAP Meatal stenosis (*n* = 1) TIP Fistula (*n* = 1) Meatal stenosis (*n* = 2)	8–80 (40)
7.	Snodgrass [12]	2006	63	4–143 (30.8)	TIP (*n *= 51) TUPU (*n* = 12) Group 1 (*n* = 44) circumcised Group 2 (*n* = 19) uncircumcised	1.6% Fistula (1)	Group 1: (1.5 -48) Group 2: (1.5 -17)
8.	Pieretti R [13]	2008	48	9–36 (15)	TIP (*n* = 40) GAP (*n* = 3) MAGPI (*n* = 5)	2% TIP Fistula (*n* = 1)	≥ 8
9.	Elbatamy AM [14]	2011	17	6–60	Modified GAP	5.9% Wound infection (*n* = 1)	6–9 (8)
10.	Bhat A et al*.* [15]	2017	11	60–288 (211.3)	TUPU (modified Duplay)	0	6–48 (30)
11.	Cendron M [16]	2018	25	NA	Duplay (*n* = 10) Modified Mathieu (*n* = 15)	8% Duplay Fistula (*n* = 1) Modified Mathieu glans Dehiscence (*n* = 1)	≥ 12
12.	Erikci V et al*.* [17]	2019	13	18–84	GAP	7.7% Fistula (*n* = 1)	6–24 (19)
13.	Duan S et al*.* [18]	2019	25	24–156 (96)	Mathieu (*n* = 5) TIP (*n* = 13) Duplay (*n* = 7)	16% Mathew: Fistula (*n* = 1) TIP: Fistula (*n* = 1) Meatal stenosis (*n* = 1) Duplay: Meatal stenosis (*n* = 1)	6–36
14.	Duan SX et al*.* [19]	2020	27	24–156 (97.5 ± 36)	TIP (*n* = 13) Duplay (*n* = 7) Mathieu (*n* = 5) MAGPI (*n* = 1) GAP (*n* = 1)	14.8 % TIP: Meatal stenosis (*n* = 1) Fistula (*n* = 1) Duplay: Meatal stenosis (*n* = 1) Mathieu: Fistula (*n* = 1)	6–36
15.	Ekberli G et al*.* [20]	2020	31	8–128 (50)	TIPU (*n* = 5) TUPU (*n* = 16) Meatoplasty (*n* = 10)	3% Duplay Fistula (*n* = 1)	Not specified
16.	Abdalla M et al*.* [21]	2022	43	12–39 (18.06 ± 6.35)	Duplay (*n* = 22) Mathieu (*n* = 21)	14% Duplay Urethral fistula (*n* = 3) Meatal stenosis (*n* = 1) Mathieu Fistula (*n* = 2)	6–23
17.	Ramaswamy R [22]	2023	12	12–87 (38.25)	TUPU (*n* = 7) TIPU (*n* = 5)	8.3% TIP: Glans necrosis (1)	1–12 (4.79)
18.	Herzberg H et al*.* [23]	2024	70	13	TIP (*n* = 40) MAGPI (*n* = 16) Duplay (*n* = 8) Mathiew (*n* = 2) GAP (*n* = 4)	22.9% Overall fistula (*n* = 11) Meatal stenosis (*n* = 5)	120

### Clinical presentation

MIP is characterized by several anatomical features distinct from the typical presentation of hypospadias. These include an intact foreskin, a wide fish mouth-like urethral opening, a deep navicular fossa, a broad shovel-shaped glans, and the absence of ventral curvature (or slight dorsal curvature of the penis).

### Surgical techniques

Management of MIP involves a variety of surgical techniques including preputial reconstruction, meatal advancement, glanuloplasty, and tubularized incised plate urethroplasty (TIP). Multiple factors influence the selection of surgical approach for MIP, including size and location of the urethral meatus, presence of penile curvature, and associated skin abnormalities. Size and location of the urethral meatus are critical determinants, with larger or more proximally positioned meatus often requiring more extensive surgical interventions to achieve optimal functional and cosmetic outcomes. If present, penile curvature necessitates consideration of techniques that address both the meatus and curvature simultaneously to ensure satisfactory correction. Furthermore, associated skin abnormalities such as the length of the foreskin or penile torsion may influence the choice of surgical technique to achieve a balance of aesthetic result and functionality. One of the early techniques used for MIP management is the pyramid procedure described by Duckett and Keating in 1989 [5]. Another core method is the glanular approximation procedure (GAP), particularly for cases with a deep glanular fissure or wide meatus. Drawbacks of GAP include possible uneven neo-urethra size and potential fistula formation. Several adjustments have been implemented over subsequent years, such as the insertion of a dorsal layer to prevent fistula formation, mobilization of glanular wings, and use of tension-free urethroplasty.

The Thiersch-Duplay or tubularized urethral plate urethroplasty (TUPU) method has demonstrated excellent functional outcomes and aesthetic results for instances with a well-developed spongiosum and wide urethral plate [18, 21]. In cases of distal hypospadias, the tubularized incised plate urethroplasty (TIP; also known as the Snodgrass technique) is highly regarded due to the low incidence of complications and positive aesthetic results [24]. This procedure entails the closure of the neourethra in two layers to minimize the risk of fistula formation. The Mathieu technique is an alternative method that conserves the distal urethral plate by flipping the proximal urethral flap to align with the urethral orifice [9]. This approach displays reduced occurrence of urethral stricture and meatal stenosis. While the Cutaneous Advancement technique has previously been employed in MIP [7], this method may not be appropriate for distal penile patients because of the very thin urethral plate in these cases. The Inframeatal Vascularized Flap Procedure [16] has also been modified in an attempt to improve patient outcomes. Overall, these various surgical techniques offer a range of options for managing different types of MIP, considering factors such as aesthetics, functionality, and potential complications.

### Outcomes

Outcome data were reported inconsistently across studies, making direct comparisons challenging. Success rates following surgical intervention ranged from 77.1 to 100%, with success defined as achievement of satisfactory cosmesis and functional outcomes. Complications were reported in 0–22.9% of cases and included meatal stenosis, urethral fistula, urinary tract infections, and cosmetic dissatisfaction (Table 2). Coincident congenital anomalies reported in MIP patients included raphe anomalies, penoscrotal web, penile chordee, undescended testes, and ureteropelvic junction obstruction. Long-term follow-up data on functional and cosmetic outcomes were limited in the available literature. In 4 studies, cosmetic evaluation was performed using PPPS and HOPE scoring systems, but no significant difference in score was noted between procedures (Table 3).

**Table 2:** Complications associated with different hypospadias procedures

Procedure	N	Fistula	Meatal stenosis	Wound dehiscence	Others
TIP	178	5	4	0	1 (glans necrosis)
MAGPI	24	0	0	0	0
Duplay	102	5	3	0	0
Mathieu	65	4	0	1	0
GAP	49	1	1	1	0
Pyramid	49	1	0	0	0
Cutaneous advancement	34	0	0	0	0
Meatoplasty	10	0	0	0	0

**Table 3:** Quality assessment of case–control studies

Question	Studies								
	Duan 2020 [19]	Cendron 2018 [16]	Bhat 2017 [15]	Duckett JW et al. [5]	Bar-Yosef [11]	Elbatamy AM [14]	Abdalla M et al. [21]	Nonomura K [9]	Erikci V et al. [17]
1.	Yes	YES	YES	YES	YES	YES	YES	YES	YES
2.	YES	YES	YES	YES	YES	YES	YES	YES	YES
3.	NO	NO	NO	NO	NO	NO	YES	NO	NO
4.	NA	YES	NA	NA	NA	NA	YES	NA	NA
5.	YES	YES	YES	YES	YES	YES	YES	YES	YES
6.	NA	NA	NA	NA	NA	NA	NA	NA	NA
7.	NA	NA	NA	NA	NA	NA	NA	NA	NA
8.	NA	NA	NA	NA	NA	NA	NA	NA	NA
9.	YES	YES	YES	YES	YES	YES	YES	YES	YES
10.	YES	YES	YES	YES	YES	YES	YES	YES	YES
11.	CD	CD	CD	CD	CD	YES	YES	CD	CD
12.	NA	NA	NA	NA	NA	NA	NA	NA	NA
Score (0–9)	5	6	5	5	5	6	8	5	5
Overall quality	Fair	Fair	Fair	Fair	Fair	Fair	Good	Fair	Fair

Question	Studies								
	Belloli GP [7]	Ramaswamy R [22]	Hill GA [8]	Ekberli G et al. [20]	Pieretti R [13]	Duan S et al. [18]	Peretz D [10]	Herzberg H et al. [23]	Snodgrass [12]
1.	YES	YES	YES	YES	YES	YES	YES	YES	YES
2.	YES	YES	YES	YES	YES	YES	YES	YES	YES
3.	NO	NO	NO	NO	NO	NO	NO	NO	NO
4.	NA	NA	NA	NA	YES	NA	NA	YES	YES
5.	YES	YES	YES	YES	YES	YES	YES	YES	YES
6.	NA	NA	NA	NA	NA	NA	NA	YES	YES
7.	NA	NA	NA	NA	NA	NA	NA	NA	NA
8.	NA	NA	NA	NA	NA	NA	NA	CD	CD
9.	YES	YES	YES	YES	YES	YES	YES	YES	YES
10.	YES	YES	YES	YES	YES	YES	YES	YES	YES
11.	CD	CD	CD	CD	CD	CD	CD	CD	CD
12.	NA	NA	NA	NA	NA	NA	NA	CD	CD
Score (0–9)	5	5	5	5	6	5	5	7	7
Overall quality	Fair	Fair	Fair	Fair	Fair	Fair	Fair	Good	Good

## Discussion

This systematic review offers a comprehensive summary of the current literature on MIP, shedding light on multiple important aspects of this rare congenital anomaly. Through a meticulous examination of epidemiological data, clinical presentations, diagnostic modalities, management strategies, and long-term outcomes, we aimed to provide a nuanced understanding of MIP in the context of clinical practice and research. A diverse array of surgical techniques was reported for the management of MIP, including preputial reconstruction, meatal advancement with glanuloplasty, tubularized incised plate urethroplasty, and various combinations thereof. Surgical approaches varied based on the severity of the condition, specific anatomical considerations, and surgeon preference.

Epidemiological data collated from diverse geographic regions indicate that MIP is a relatively uncommon condition, with incidence rates varying significantly between populations. However, the true prevalence may be underestimated due to underreporting or misdiagnosis, emphasizing the need for heightened clinical awareness and standardized diagnostic criteria [25, 26]. Our review highlights considerable heterogeneity in the clinical presentation of MIP, which spans a spectrum from isolated widening of the urethral meatus to cases that also feature penile curvature, undescended testes, or concurrent penile skin abnormalities [27]. MIP is distinguished from the typical manifestation of hypospadias by the presence of an intact foreskin, urethral opening resembling a wide fish mouth, a deep navicular fossa, a broad shovel-shaped glans, and the lack of downward curvature (or minor upward curve of the penis) [28–31].

MIP is often identified during neonatal circumcision or later in life when the prepuce is retracted in non-circumcised boys. Clinical presentation can vary; some patients may have a mildly dilated meatal opening, while others may present with a large, fish mouth-shaped opening that extends to or just below the coronal margin. In cases where the meatus is more enlarged, patients may report a splayed urinary stream, but no other urologic anomalies have been associated with this variant. In the absence of other symptoms, radiologic evaluation is not indicated.

It is important to recognize that MIP may not always be associated with functional issues, and there is no evidence of penile curvature associated with this variant. As such, artificial erection testing during repair is not recommended. Surgical repair is typically considered for patients presenting with a significantly large, fish-mouth or blunderbuss-shaped meatus that opens near or at the coronal margin. The decision to operate is based on clinical presentation and the potential for long-term functional or cosmetic issues. Non-surgical management may be appropriate in cases with only slight enlargement of the urethral opening, since previous studies have not documented any adverse outcomes in this group. However, further follow-up is required to determine the long-term consequences if surgery is deferred.

Our review uncovered a range of different management strategies, reflecting a lack of consensus or standardized guidelines for MIP. Surgical intervention, ranging from meatoplasty to more complex reconstructive procedures, is often indicated to address functional and cosmetic concerns, particularly for obstructive symptoms, urinary spraying, or sexual dysfunction. There is ongoing debate about the benefits of operating on MIP patients given the unique surgical difficulties that these patients present. Indeed, surgical management of MIP is complicated by the fact that the majority of affected patients have undergone circumcision. This results in thin, scarred penile skin that is devoid of preputial and dartos tissue, which are essential components for reconstructive procedures. While Snodgrass' research demonstrated that circumcision has no bearing on TIPU outcomes in a subset of patients with MIP [12, 32]**,** the anatomical attributes of this hypospadias variant pose an exceptional challenge for surgical professionals. Glanular wings may become thinner when the urethral plate and broad meatus are separated, increasing the likelihood that they will rupture and develop a fistula. Due to dissatisfaction with perimeatal-based flap and meatal advancement and glanuloplasty (MAGPI) techniques, Duckett and Keating [5] devised the ‘pyramid’ method, which aims to address the issues of a large glanular fissure with non-compliant urethral opening via the addition of a glans approximation procedure (GAP). Unlike the GAP and pyramid procedures, which involve dissecting lateral aspects of the urethral plate, the TIPU technique permits a more thorough dissection resulting in broader glanular wings more suitable for reconstruction. Nonetheless, optimal timing, technique, and long-term outcomes of surgical interventions for MIP remain unclear and require further prospective studies with long-term follow-up.

Surgical correction of MIP presents unique difficulties due to wide splaying of the distal urethra and the absence of spongiosum, which renders the ventral portion of the urethral meatus composed of extremely thin, relatively immobile tissue [33, 34]. Accordingly, Bar-Yosef [11] routinely combine elements of TIP, urethroplasty, and the ‘pyramid’ procedure, tailoring their technique to the anatomical characteristics of each patient. Indeed, when a surgeon endeavors to rectify hypospadias in a circumcised boy, they may encounter further complexities arising from restricted access to dartos tissue and local fibrosis caused by secondary healing processes. Duan et al. [18] observed that there is no statistically significant variation in operation time when comparing Mathieu, TIP, and Duplay techniques. Furthermore, there was no significant difference between the three methods in terms of intraoperative hemorrhage, length of hospital stay, rate of postoperative analgesia, or rate of cure (*P* > 0.05). Success rates associated with Mathieu, TIP, and Duplay were initially estimated to be 80%, 84.6%, and 85.7%, respectively. Urinary fistula development was observed as early as 2 weeks post-surgery, and meatal stenosis was typically identified at ~ 48 weeks post-surgery.

Long-term outcomes and quality-of-life assessments for individuals with MIP are relatively sparse in the literature, highlighting a critical gap in current knowledge. While surgical interventions aim to improve urinary function, cosmesis, and sexual outcomes, several potential complications remain including urethral strictures, fistula formation, and recurrent meatal stenosis. Herzberg et al. [22] conducted a study on the long-term results of 70 patients with MIP. Median follow-up period was 10 years, with an interquartile range of 6–13 years, with 32% of cases later undergoing a second procedure. Univariate analysis performed on the MIP hypospadias group revealed no correlation between reoperation and initial patient features, such as the existence of ventral curvature or meatal location.

This systematic review provides valuable new insight into the clinical management of MIP but still features several limitations. The inherent biases and variability in study designs, sample sizes, and reporting practices across included studies may influence the generalizability and robustness of our findings. Moreover, the retrospective nature of many studies precludes causal inference and limited our ability to assess temporal trends or treatment outcomes longitudinally. Furthermore, publication bias may skew the available evidence towards more severe or atypical cases, potentially underrepresenting milder forms of MIP managed conservatively in clinical practice.

In light of these limitations, future research efforts should prioritize prospective multicenter studies with standardized diagnostic criteria and outcome measures to elucidate the natural history, optimal management strategies, and long-term outcomes of MIP. Collaborative initiatives, such as international registries or consortia, may facilitate data sharing, enhance statistical power, and accelerate progress towards evidence-based guidelines for MIP management. Furthermore, advances in imaging modalities, such as 3D reconstruction techniques [35], and functional imaging [36], hold considerable promise for improving preoperative planning and surgical outcomes in MIP. Performing a 3D scan during hypospadias surgery enables precise measurement of the width and length of the urethral plate, as well as penile shaft length and glans width. This measurement method has a high level of consistency among different evaluators, which helps establish a reliable comparison of phenotypes and outcomes of various surgical procedures [35]. Indeed, Abbas et al. [36] demonstrated that image recognition and machine learning algorithms can be used to standardize urethral plate evaluation by scoring hypospadias photos.

In conclusion, this systematic review provides a comprehensive overview of megameatus intact prepuce, highlighting clinical heterogeneity, diagnostic challenges, management strategies, and long-term outcomes. Given the psychosocial impact of MIP on affected individuals and their families, careful postoperative monitoring and longer post-pubertal evaluation are crucial. While significant strides have been made in understanding and managing MIP, critical gaps in knowledge persist, necessitating continued research efforts, assessment of patient-reported outcomes, and multidisciplinary qualitative studies. By addressing these knowledge gaps and standardizing clinical practice, we can optimize outcomes and quality of life for individuals affected by this rare congenital anomaly.

## Data Availability

No datasets were generated or analysed during the current study.
